# Perceived Organizational Support and Nurses’ Pressure Injury Care Behaviors: The Serial Mediating Roles of Self‐Efficacy and Change Fatigue

**DOI:** 10.1155/jonm/5681859

**Published:** 2026-10-15

**Authors:** Chenxin Zhang, Yi Liu, Zhenghui Dong

**Affiliations:** ^1^ School of Nursing Xinjiang Medical University Urumqi 830017 Xinjiang China xjmu.edu.cn; ^2^ The Sixth Affiliated Hospital of Xinjiang Medical University Urumqi 830002 Xinjiang China xjmu.edu.cn; ^3^ Health Care Research Center for Xinjiang Regional Population Urumqi 830017 Xinjiang China

**Keywords:** change fatigue, nursing care behaviors, path analysis, perceived organizational support, pressure injury, self-efficacy, serial mediation model

## Abstract

**Aim:**

To examine the relationship between perceived organizational support and nurses’ pressure injury care behaviors and to test the serial mediating roles of self‐efficacy in pressure injury management and change fatigue.

**Background:**

Pressure injuries are largely preventable, and nurses’ care behaviors are critical to prevention. Perceived organizational support may influence these behaviors, but the mediating mechanisms remain unclear.

**Methods:**

From November 2025 to June 2026, 524 nurses from three tertiary‐level A hospitals in Xinjiang were recruited using purposive sampling. Data were collected using the Perceived Organizational Support Scale, the Pressure Ulcer Management Self‐Efficacy Scale, the Change Fatigue Scale, and the Pressure Injury Care Behavior Questionnaire. Path analysis was conducted using AMOS 26.0, with serial mediation tested using bias‐corrected bootstrapping (5000 resamples).

**Results:**

Perceived organizational support was positively associated with self‐efficacy in pressure injury management (*β* = 0.590, *p* < 0.001) and pressure injury care behaviors (*β* = 0.387, *p* < 0.001). Self‐efficacy was negatively associated with change fatigue (*β* = −0.268, *p* < 0.001), and change fatigue was negatively associated with pressure injury care behaviors (*β* = −0.187, *p* < 0.001). The serial indirect effect was 0.021 (95% CI: 0.012–0.032).

**Conclusions:**

The findings are consistent with a serial indirect pathway in which perceived organizational support is associated with nurses’ pressure injury care behaviors through self‐efficacy and change fatigue. Longitudinal studies are needed to confirm this pathway.

**Implications for Nursing Management:**

Nursing managers should strengthen organizational support, cultivate nurses’ self‐efficacy in pressure injury management, and monitor change fatigue during organizational change to support preventive care behaviors.

## 1. Introduction

Pressure injuries (PIs) represent one of the most common iatrogenic skin injuries encountered in clinical nursing, prolonging hospital stays and increasing infection and mortality risks [1]. Because PIs are largely preventable, their prevention serves as a direct indicator of healthcare quality and safety [2]. In 2021, global PI incidence among individuals aged over 60 reached 1.725 million cases, reflecting a 155% rise from 1990 [1]. By the end of 2023, China had over 217 million people aged 65 and above, a population particularly vulnerable to PIs due to age‐related skin degeneration [2]. Preventing PIs depends heavily on nurses’ adherence to standardized care behaviors, including risk assessment, skin inspection, repositioning, and prophylactic dressing use. Exploring the determinants of these behaviors is therefore crucial for improving nursing quality and advancing global patient safety goals.

Nurses, as frontline practitioners, directly influence PI incidence through their care behaviors. However, they encounter obstacles including knowledge gaps, excessive workloads, resource shortages, and inadequate organizational backing. A survey of Jordanian ICU nurses identified leadership support as a key factor promoting guideline compliance [3], while a Hungarian national survey highlighted managerial commitment and multidisciplinary collaboration [4]. These findings indicate that organizational‐level factors may be more proximal determinants of nurses’ PI care behaviors than individual knowledge or attitudes alone. However, the mechanisms through which organizational resources translate into preventive behaviors remain unclear.

Perceived organizational support (POS) refers to employees’ perception of the extent to which their organization values their contributions and cares about their well‐being. Within occupational health research, POS has been consistently linked to employees’ health‐promoting preventive actions [5–7]. However, whether and how POS influences nurses’ specific clinical preventive practices—particularly PI care behaviors—remains empirically underexamined. This gap is important because POS may operate not only directly but also through intermediate psychological and occupational processes.

According to the Job Demands–Resources (JD‐R) model [8], job resources foster positive outcomes by enhancing engagement, while job demands deplete psychological resources. Complementing this, Conservation of Resources (COR) theory posits that individuals are motivated to protect and acquire resources, with initial gains facilitating further resource accumulation—a “gain spiral” [9]. Within these frameworks, POS can be regarded as an external job resource, whereas self‐efficacy constitutes a vital internal psychological resource [10]. Complementary evidence from organizational behavior research suggests that organizational or behavioral factors can be transmitted to individual behavior through self‐efficacy [11], and that organizational support theory helps explain how organizational environmental factors are converted into individual behavior [12]. Although these studies were conducted in non‐nursing contexts, they provide mechanistic support for the role of self‐efficacy in transferring the effect of the organizational environment to professional behavior. Together, these theoretical perspectives suggest a resource conversion pathway in which organizational support is internalized as self‐efficacy, which in turn buffers change fatigue and ultimately promotes preventive care behavior. In preventive behavior research, self‐efficacy has been established as a significant driver of health‐related actions [13]. However, change fatigue—a state of exhaustion arising from frequent organizational changes [14]—may erode this psychological resource. When nurses experience high change fatigue, their attentional resources are depleted, potentially undermining preventive behavior implementation [15]. Whether self‐efficacy and change fatigue serially mediate the POS–PI care behavior relationship has not been empirically tested.

Grounded in the JD‐R model and COR theory, we hypothesize that POS directly facilitates preventive behaviors (H1) and enhances self‐efficacy (H2); self‐efficacy reduces change fatigue (H3); change fatigue impairs preventive behaviors (H4); self‐efficacy directly drives preventive behaviors (H6); POS reduces change fatigue (H7); and self‐efficacy and change fatigue serially mediate the POS–behavior relationship (H5). The conceptual model depicting these hypothesized paths is presented in Figure 1.

**FIGURE 1:**
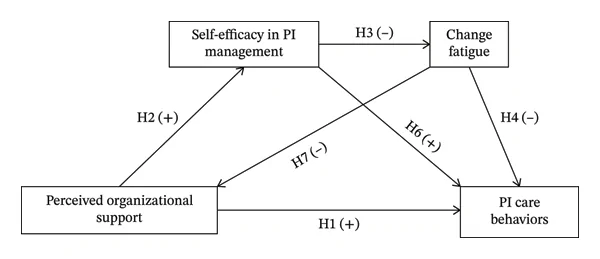
Conceptual model of the hypothesized serial mediation pathway. Note: H5 represents the serial mediation effect through self‐efficacy in PI management and change fatigue. PI = pressure injury; POS = perceived organizational support.

This study aims to examine the serial mediation pathway linking POS, self‐efficacy in PI management, change fatigue, and PI care behaviors among clinical nurses. Specifically, it addresses three research questions: (1) Is POS associated with nurses’ PI care behaviors? (2) Does self‐efficacy in PI management mediate this association? (3) Does change fatigue further mediate the relationship between self‐efficacy and PI care behaviors, forming a serial indirect pathway? The findings are expected to provide a theoretical basis for nursing managers to optimize PI prevention strategies and to inform occupational health protection during organizational change.

## 2. Materials and Methods

### 2.1. Participants

From November 2025 to June 2026, a purposive sampling strategy was employed to recruit wound care specialists and frontline clinical nurses from three tertiary‐level A hospitals in Xinjiang, China. Eligibility criteria were as follows: (1) current employment in a tertiary‐level A hospital in Xinjiang with a valid nursing practice license and (2) voluntary participation with signed informed consent. Exclusion criteria were as follows: (1) nurses undergoing advanced training, standardized residency training, or internship during the survey period and (2) those concurrently participating in other comparable nursing intervention studies. Of the 540 questionnaires distributed, 524 were deemed valid (97.04%). Of these, 199 (38.0%) were from Hospital A, 168 (32.1%) from Hospital B, and 157 (30.0%) from Hospital C. All participants provided informed consent, and the study protocol was approved by the Ethics Committee of the Sixth Affiliated Hospital of Xinjiang Medical University (approval number: LFYLLSC20251219‐011).

### 2.2. Instruments

#### 2.2.1. General Information Questionnaire

A self‐designed questionnaire was used to collect sociodemographic and occupational data, including gender, age, highest educational attainment, professional title, nurse level, years of service, and frequency of caring for patients with hospital‐acquired PIs over the preceding 2 years. Data collection was carried out on‐site by two trained investigators.

#### 2.2.2. Nurses’ POS Scale

This scale, revised by Zuo et al. [16], comprises 13 items rated on a 5‐point Likert scale, with higher total scores reflecting greater POS. The original scale reported a Cronbach’s *α* of 0.92 [16]. In the present sample, *α* was 0.993; this exceeds the original value and may reflect sample homogeneity or item redundancy, so it should be interpreted with caution.

#### 2.2.3. Chinese Version of the Change Fatigue Scale (CFS)

Originally developed by Bernerth [17]and culturally adapted by Zhang et al. [18], the CFS contains six items scored on a 7‐point Likert scale; higher scores indicate more pronounced change fatigue. Cronbach’s *α* in this study was 0.962, indicating outstanding internal consistency.

#### 2.2.4. Chinese Version of the Pressure Ulcer Management Self‐Efficacy Scale for Nurses (PUM‐SES)

This scale was originally developed by Dellafiore et al. [19] and subsequently translated into Chinese by Hou et al. [20]. It includes 10 items rated on a 5‐point Likert scale, with higher scores denoting greater self‐efficacy. Cronbach’s *α* was 0.986 in this investigation.

#### 2.2.5. PI Care Behavior Questionnaire

Designed by Li et al. [21], this questionnaire consists of 14 items on a 5‐point Likert scale, with total scores ranging from 14 to 70; higher scores reflect better care behaviors. Cronbach’s *α* for this instrument was 0.941.

### 2.3. Statistical Analysis

Data were entered and analyzed using SPSS 26.0 for descriptive statistics and correlation analyses. Categorical variables were summarized as frequencies and percentages. Continuous variables, which deviated from normality according to the Kolmogorov–Smirnov test (*p* < 0.05), were presented as means ± standard deviations. Common‐method variance was assessed using Harman’s single‐factor test and a single‐factor confirmatory factor analysis. Given the large sample (*N* = 524) and acceptable skewness and kurtosis, means and standard deviations were used, with median and interquartile range additionally reported in Table 1. Skewness ranged from −1.500 to −0.313 and kurtosis from −1.039 to 3.913, within acceptable thresholds (|skewness| < 2, |kurtosis| < 7). Mardia’s test indicated significant multivariate skewness (b1p = 6.58, *p* < 0.001) and kurtosis (b2p = 37.03, *p* < 0.001). ML estimation was retained given the adequate sample size (*N* = 524), and the indirect effect was tested using bias‐corrected bootstrapping (5000 resamples), which does not rely on normality. As a robustness check, the model was re‐estimated using the MLM estimator; all path coefficients and the serial indirect effect were identical to those obtained with standard ML. Correlations among variables were assessed using Spearman’s rank correlation. Path analysis was performed with AMOS 26.0 using maximum likelihood estimation based on the covariance matrix; means and intercepts were not estimated. The hypothesized model is a saturated recursive path model containing four observed variables and six freely estimated structural paths: POS ⟶ self‐efficacy, POS ⟶ change fatigue, POS ⟶ PI care behaviors, self‐efficacy ⟶ change fatigue, self‐efficacy ⟶ PI care behaviors, and change fatigue ⟶ PI care behaviors. The model had 10 free parameters: six structural paths, three disturbance variances for the endogenous variables, and one variance for the exogenous variable (POS). The sample covariance matrix provided 10 distinct sample moments: four variances and six covariances. Therefore, the model is just‐identified (df = 0); conventional fit indices (CFI, TLI, RMSEA, and SRMR) are therefore not defined. Model evaluation relied on the significance of path coefficients and bootstrap confidence intervals (CIs). As a supporting check, an over‐identified model constraining the two paths corresponding to hypotheses H7 (POS ⟶ change fatigue) and H6 (self‐efficacy ⟶ PI care behaviors) to zero was estimated (df = 2); this constrained model showed poor fit (*χ* ^2^ = 265.590, CFI = 0.763, TLI = 0.289, RMSEA = 0.502, SRMR = 0.101), confirming that these two paths are nontrivial and that the saturated specification is appropriate. In addition, a sensitivity analysis was conducted in which years of service, frequency of caring for patients with hospital‐acquired PIs in the past 2 years, and hospital site were entered as covariates predicting self‐efficacy in PI management, change fatigue, and PI care behaviors. The significance of mediation effects was tested using bias‐corrected bootstrapping with 5000 resamples. A 95% CI that did not include zero was considered statistically significant. Direct, indirect, and total effects are reported as unstandardized coefficients; standardized path coefficients are additionally reported for individual paths in Table 2. This reporting format follows contemporary recommendations for presenting indirect effects and CIs in mediation analysis [22]. The significance level was set at *α* = 0.05 (two‐tailed).

**TABLE 1:** Descriptive statistics of the study variables (*N* = 524).

Variable	No. of items	Possible range	Mean ± SD	Median (IQR)	Skewness	Kurtosis
Change fatigue	6	6–42	27.12 ± 10.26	29.00 (18.75–36.00)	−0.313	−1.039
Perceived organizational support	13	13–65	47.73 ± 13.35	49 (39–61)	−0.597	−0.309
Self‐efficacy in PI management	10	10–50	35.64 ± 9.97	37 (30–42)	−0.438	−0.295
PI care behaviors	14	14–70	57.53 ± 9.66	58 (53–65)	−1.500	3.913

**TABLE 2:** Path coefficients of the serial mediation model.

Path	Unstandardized coefficient	Standardized coefficient (*β*)	S.E.	C.R.	*p*
Perceived organizational support ⟶ self‐efficacy in PI management	0.441	0.590	0.032	13.885	< 0.001
Self‐efficacy in PI management ⟶ change fatigue	−0.275	−0.268	0.057	−4.842	< 0.001
Change fatigue ⟶ PI care behaviors	−0.176	−0.187	0.023	−7.569	< 0.001
Perceived organizational support ⟶ PI care behaviors	0.280	0.387	0.033	8.600	< 0.001
Self‐efficacy in PI management ⟶ PI care behaviors	0.448	0.462	0.042	10.684	< 0.001
Perceived organizational support ⟶ change fatigue	−0.235	−0.305	0.043	−5.478	< 0.001

*Note:* The model was saturated (df = 0), estimated from the covariance matrix without means/intercepts. It had 10 free parameters (six structural paths, three disturbance variances, and one exogenous variance) and 10 sample moments (four variances and six covariances). Because free parameters equal sample moments, overall fit indices are not defined. An over‐identified model (df = 2) constraining POS ⟶ change fatigue and self‐efficacy ⟶ PI care behaviors to zero showed poor fit (CFI = 0.763, RMSEA = 0.502, SRMR = 0.101), supporting the saturated model. Both unstandardized and standardized coefficients are reported; S.E., C.R., and P refer to unstandardized estimates. Direct, indirect, and total effects in Table 5 are unstandardized.

## 3. Results

### 3.1. Participant Characteristics

Of the 524 participating nurses, the majority were female (95.6%), held a bachelor’s degree (87.6%), and had a professional title of senior nurse or above (84.2%). Furthermore, 87.6% had cared for patients with hospital‐acquired PIs in the preceding 2 years. Detailed demographic and occupational characteristics are presented in Table 3.

**TABLE 3:** Demographic and occupational characteristics of the participants (*N* = 524).

Variable	Category	*n*	Percentage (%)
1. Gender	Male	23	4.4
	Female	501	95.6

2. Age (years)	≤ 25	24	4.6
	25–30	135	25.8
	31–35	162	30.9
	36–40	139	26.5
	41–45	50	9.5
	≥ 46	14	2.7

3. Highest education	Associate degree	58	11.1
	Bachelor’s degree	459	87.6
	Master’s or above	7	1.3

4. Professional title	Junior nurse	75	14.3
	Senior nurse	241	46
	Supervisor nurse	188	35.9
	Associate senior or above	20	3.8

5. Nurse level	N0	22	4.2
	N1	93	17.7
	N2	196	37.4
	N3	191	36.5
	N4	22	4.2

6. Length of service (years)	≤ 5	85	16.2
	6–10	168	32.1
	11–15	133	25.4
	16–20	97	18.5
	≥ 21	41	7.8

7. Frequency of caring for patients with hospital‐acquired PIs in the past 2 years	Often (≥ 5 cases)	249	47.5
	Occasionally (1–4 cases)	254	48.5
	Never	21	4

### 3.2. Multicollinearity Diagnostics

Variance inflation factor (VIF) values were computed to assess multicollinearity among the predictor variables. The VIFs for POS, self‐efficacy in PI management, and change fatigue were 1.66, 1.63, and 1.35, respectively, all well below the threshold of 5, indicating no significant multicollinearity concerns.

### 3.3. Descriptive Statistics of the Study Variables

The mean scores for the sample were as follows: POS, 47.73 ± 13.35; self‐efficacy in PI management, 35.64 ± 9.97; change fatigue, 27.12 ± 10.26; and PI care behaviors, 57.53 ± 9.66 (Table 1). Common‐method variance. Harman’s single‐factor test showed that the first factor accounted for 34.76% of the variance, below the 40% threshold. A single‐factor confirmatory factor analysis showed poor fit (CFI = 0.310, TLI = 0.275, RMSEA = 0.204, SRMR = 0.152), indicating that a single common method factor did not adequately explain the data. Skewness ranged from −1.500 to −0.313 and kurtosis from −1.039 to 3.913.

### 3.4. Correlation Analysis

Spearman correlation analysis (Table 4) revealed that POS was positively correlated with self‐efficacy in PI management (*r* = 0.533) and PI care behaviors (*r* = 0.718), and negatively correlated with change fatigue (*r* = −0.482). Self‐efficacy in PI management was negatively correlated with change fatigue (*r* = −0.473) and positively correlated with PI care behaviors (*r* = 0.767). Change fatigue was negatively associated with PI care behaviors (*r* = −0.646). All correlation coefficients were statistically significant (all *p* < 0.001).

**TABLE 4:** Spearman correlation matrix among the study variables (*N* = 524).

Variable	Change fatigue	Perceived organizational support	Self‐efficacy in PI management	PI care behaviors
Change fatigue	1			
Perceived organizational support	−0.482^∗∗^	1		
Self‐efficacy in PI management	−0.473^∗∗^	0.533^∗∗^	1	
PI care behaviors	−0.646^∗∗^	0.718^∗∗^	0.767^∗∗^	1

^∗∗^ *p* < 0.001.

### 3.5. Path Analysis and Serial Mediation Effect Testing

The hypothesized model is a saturated recursive path model containing four observed variables and six freely estimated structural paths. The model had 10 free parameters (six structural paths, three disturbance variances, and one exogenous variance) and 10 distinct sample moments (four variances and six covariances). Therefore, the model is just‐identified (df = 0); traditional overall fit indices (CFI, TLI, RMSEA, and SRMR) are therefore not defined. An over‐identified model constraining the two paths corresponding to H7 and H6 to zero (df = 2) showed poor fit (*χ* ^2^ = 265.590, CFI = 0.763, TLI = 0.289, RMSEA = 0.502, and SRMR = 0.101), confirming that these paths should be retained and that the saturated specification is appropriate. The evaluation of this model is primarily based on the significance of path coefficients and the Bootstrap CIs for mediating effects. Given that the Bootstrap method is one of the most robust methods for testing indirect effects, this study employs the bias‐corrected percentile Bootstrap method (with 5000 repetitions) to test for mediation. If the 95% CI does not include 0, the mediation effect is considered significant.

Sensitivity analysis adjusting for professional background and hospital site. Hospital B had lower mean scores for POS (37.16 vs. 52.15 and 53.45), self‐efficacy (27.21 vs. 39.05 and 40.34), and PI care behaviors (48.11 vs. 61.17 and 63.01), and higher change fatigue (33.29 vs. 24.82 and 23.43). After adding years of service, PI‐care experience, and hospital site as covariates, all six focal paths remained significant, though attenuated: POS ⟶ self‐efficacy (*β* = 0.383), POS ⟶ change fatigue (*β* = −0.260), self‐efficacy ⟶ change fatigue (*β* = −0.207), POS ⟶ PI care behaviors (*β* = 0.332), self‐efficacy ⟶ PI care behaviors (*β* = 0.386), and change fatigue ⟶ PI care behaviors (*β* = −0.163), all *p* < 0.001. The serial indirect effect remained significant (0.009, *p* = 0.001). Hospital site was significantly associated with self‐efficacy, change fatigue, and PI care behaviors (Hospital B vs. Hospital A: *β* = −0.351, 0.142, and −0.179, respectively).

The path coefficients are presented in Table 2. POS positively predicted self‐efficacy for pressure ulcer management (*β* = 0.590, *p* < 0.001) and pressure ulcer care behaviors (*β* = 0.387, *p* < 0.001); self‐efficacy for pressure ulcer management positively predicted pressure ulcer care behaviors (*β* = 0.462, *p* < 0.001); POS negatively predicted change fatigue (*β* = −0.305, *p* < 0.001); self‐efficacy for pressure ulcer management negatively predicted change fatigue (*β* = −0.268, *p* < 0.001); and change fatigue negatively predicted pressure ulcer care behaviors (*β* = −0.187, *p* < 0.001). All direct paths were statistically significant. The final path model with standardized coefficients is shown in Figure 2.

**FIGURE 2:**
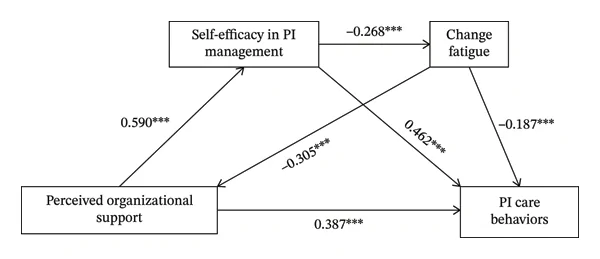
Path diagram of the serial mediation model. Note. Values shown are standardized path coefficients. The model is a saturated recursive path model (df = 0); all six structural paths and disturbance variances were freely estimated. Means and intercepts were not estimated. ^∗∗∗^ *p* < 0.001.

The bootstrap results (Table 5) indicated that the serial indirect effect along the pathway “POS ⟶ self‐efficacy in PI management ⟶ change fatigue ⟶ PI care behaviors” was 0.021 (95% CI: 0.012–0.032), with the CI excluding zero, indicating a significant statistical indirect association. As a robustness check, the MLM estimator produced identical path coefficients and serial indirect effect to standard ML.

**TABLE 5:** Unstandardized bootstrap results for the serial mediation effect.

Path	Direct effect (unstandardized, 95% CI)	Indirect effect (unstandardized, 95% CI)	Total effect (unstandardized, 95% CI)
Perceived organizational support⟶ Self‐efficacy ⟶ Change fatigue ⟶ PI care behaviors	0.280^∗∗∗^ (0.222–0.351)	0.021^∗∗∗^ (0.012–0.032)	0.540^∗∗∗^ (0.476–0.606)

*Note:* All effects are unstandardized coefficients based on bias‐corrected bootstrapping with 5000 resamples. The indirect effect represents a statistical indirect association and should not be interpreted as a causal effect. Standardized path coefficients are reported separately in Table 2.

^∗∗∗^ *p* < 0.001.

## 4. Discussion

### 4.1. Integration of the JD‐R Model and COR Theory: Findings Consistent With a “Resource Conversion–Depletion Buffering–Behavior Promotion” Chain

To our knowledge, this study is among the first to empirically examine the complete serial pathway “POS ⟶ self‐efficacy in PI management ⟶ change fatigue ⟶ PI care behaviors” within the PI prevention literature. The findings are statistically consistent with this serial pathway (indirect effect = 0.021, 95% CI excluding zero) and suggest that the association between POS and PI care behaviors may operate through the sequential intermediate variables of self‐efficacy and change fatigue. Because of the cross‐sectional design, this pattern should be interpreted as a statistical indirect association rather than a demonstrated temporal or causal sequence [9, 23]. Importantly, the focal associations were robust to adjustment for years of service and prior PI‐care experience, suggesting that the observed POS–behavior association is not simply a reflection of nurses’ experience or professional position.

Compared with prior research, this study makes three theoretical contributions. First, whereas previous JD‐R applications have treated the motivational and health‐impairment processes as parallel, our findings suggest they may be sequentially linked: external resources (POS) are associated with internal resources (self‐efficacy), which are associated with reduced depletion (change fatigue) and ultimately with preventive behavior. Second, we operationalize COR theory’s “gain spiral” as a testable variable‐level chain. Third, we extend change fatigue from organizational behavior into the patient safety domain as a potential upstream risk factor for preventable injuries.

Prior applications of the JD‐R model have often treated the motivational pathway (job resources ⟶ work engagement ⟶ positive outcomes) and the health‐impairment pathway (job demands ⟶ burnout ⟶ negative outcomes) as parallel and independent processes [8]. Our serial mediation model suggests a possible sequential linkage between them: the data are consistent with the possibility that external resources are associated with reduced depletion via internal resources, which in turn is associated with more positive behaviors. The first segment of the chain provides empirical support for the conversion of external into internal resources, a core tenet of the extended JD‐R model; the second segment provides empirical support for the inhibition of depletion by internal resources, a central mechanism of COR’s gain spiral. These two theoretical strands converge at self‐efficacy in PI management, forming a coherent “resource conversion–depletion buffering–behavior promotion” chain. Hobfoll [9]emphasized that resource loss exerts a stronger impact on individuals than resource gain; our findings further suggest that the “conversion efficiency” of resources may hold greater predictive value for behavior than their absolute quantity. This integrated framework extends the JD‐R model’s scope by proposing that, when examining nurses’ preventive behaviors, resource “gain” and “loss” should not be viewed in isolation but rather in terms of their reciprocal conversion and transmission. The substantial between‐hospital differences in POS, self‐efficacy, change fatigue, and PI care behaviors highlight the role of organizational‐level factors in shaping nurses’ preventive behaviors.

Viewed through the lens of the social‐ecological model, our serial pathway maps neatly onto a multitiered influence chain: organizational level ⟶ individual psychological level ⟶ occupational health level ⟶ behavioral level [24]. This model posits that individual behavior is nested within interconnected environmental systems, with dynamic interactions across levels. Our pathway empirically supports this premise—organizational resource provision activates individual belief systems, which in turn alleviate occupational depletion, ultimately steering behavioral outcomes. This alignment suggests that PI prevention interventions should target not a single tier but rather a comprehensive framework that integrates organizational support, psychological empowerment, and occupational health surveillance. Suo et al. [23] reported that POS was directly associated with work engagement among newly recruited nurses, and also operated indirectly via self‐efficacy, echoing our methodological approach. The distinctive contribution of our study lies in extending the endpoint from generic “work performance” to “PI care behaviors”—a clinically meaningful patient safety indicator—thereby conferring direct translational value to the theoretical model. Based on Bandura’s social cognitive theory and the JD‐R model, this study expanded the initial model by adding two pathways—“self‐efficacy ⟶ nursing behavior” and “perceived organizational support ⟶ change fatigue”—to more comprehensively reflect the mechanisms through which organizational resources influence clinical behavior via the dual pathways of psychological capital and professional burnout.

### 4.2. Direct Effect of POS on Care Behaviors: The “Efficacy Spillover” of External Resources

Our results revealed that POS was significantly and positively associated with PI care behaviors (*β* = 0.387, *p* < 0.001), with this direct effect accounting for approximately 51.9% of the total effect. This suggests that, within this cross‐sectional sample, the association between POS and nurses’ preventive actions is predominantly direct—when nurses perceive robust organizational support in terms of staffing, training, equipment, and emotional care, their adherence to and proactiveness in executing preventive measures increase correspondingly [25]. This finding lends strong empirical support to the extended JD‐R model [8]. Suo et al. [23]similarly found that POS directly predicted work engagement. Our study further clarifies that within the highly standardized context of PI prevention, the effect of POS on behavior is primarily direct—external resource provision translates directly into the conditional guarantees necessary for behavioral execution, rather than being mediated entirely through individual psychological beliefs.

Traditional PI prevention research has often relied on the Health Belief Model or the Knowledge‐Attitude‐Practice (KAP) framework, portraying preventive behaviors as rational decisions shaped by individual risk perception, severity appraisal, and benefit evaluation. In contrast, our findings indicate that in the highly institutionalized hospital environment, nurses’ preventive behaviors may better be characterized as “organizationally enabled normative execution” rather than purely “individual cognition‐driven rational choice.” When organizations supply adequate training, reasonable scheduling, sufficient equipment, and clear protocols, the execution of preventive measures gains institutional support. Even when individual nurses vary in risk perception, behavioral standardization can be upheld through organizational process constraints [26]. This implies that to systematically reduce PI incidence, interventions should not be confined to individual knowledge updates or attitudinal modifications but should preferentially address organizational‐level resource assurance and environmental optimization. The direct effect of self‐efficacy on nursing behavior (*β* = 0.462) remained significant even after controlling for the chain path, suggesting that self‐efficacy is not only a mediating factor but also an independent driver of behavior; therefore, organizational empowerment must be balanced with the cultivation of individual beliefs.

### 4.3. Self‐Efficacy as a “Resource Converter”: Cognitive Transformation From External Resources to Internal Capital

Our study found that POS was positively associated with self‐efficacy (*β* = 0.590, *p* < 0.001), and self‐efficacy was negatively associated with change fatigue (*β* = −0.268, *p* < 0.001). This corroborates the central proposition of Bandura’s social cognitive theory—that self‐efficacy constitutes the key cognitive mechanism by which individuals translate external environmental resources into internal psychological capital [10]. Bandura argued that beliefs about personal capabilities not only directly influence behavioral choices and persistence but also shape how individuals interpret and respond to environmental cues, which precisely underpins the “internalization” of POS as an external resource.

From the COR perspective, this finding illuminates the initiation of the “resource gain spiral.” COR theory holds that resource acquisition can set off a gain spiral, wherein initial gains facilitate further resource accumulation [9]. Our serial pathway is consistent with this initiation process: higher POS was associated with greater self‐efficacy, and greater self‐efficacy was in turn associated with lower change fatigue. This translates the abstract concept of the “gain spiral” into an empirically testable variable‐level chain of associations [27]. Ni et al. [28] confirmed that POS positively predicts general self‐efficacy in nurses. Collectively, these findings suggest that self‐efficacy plays a pivotal “resource converter” role between POS and change fatigue—organizational resources need to be transformed into individual internal beliefs to fully exert their buffering effect against psychological depletion [27]. In the absence of this conversion step, the supply of external resources may be filtered by the individual’s cognitive system and fail to realize its full potential to counteract depletion. The direct inhibitory effect of POS on change fatigue (*β* = −0.305) does not depend entirely on self‐efficacy as a mediator, suggesting that improving objective working conditions alone can produce an independent stress‐reducing effect.

### 4.4. Depletion Effect of Change Fatigue on Preventive Behaviors: Attentional Resource Exhaustion and the Insidious Erosion of Patient Safety

Our results showed that change fatigue was negatively associated with PI care behaviors (*β* = −0.187, *p* < 0.001), meaning that higher levels of change fatigue were associated with poorer compliance and execution quality in PI prevention. This finding is consistent with the applicability of the JD‐R model’s health‐impairment pathway to the domain of nursing preventive behaviors [8].

Change fatigue refers to the sustained stress experienced by nurses due to rapid and continuous organizational changes in the workplace, leading to negative states such as mental exhaustion, increased burnout, and decreased work engagement [29]. From the perspective of patient safety science, the essence of change fatigue is a “state of attention resource depletion”—when nurses are in a state of psychological resource depletion due to frequent organizational changes, their working memory capacity, executive function, and risk monitoring abilities are all impaired. In his theoretical framework on medical errors, Reason [15] points out that the depletion of attentional resources is a core precursor to procedural lapses. Change fatigue erodes the quality of nursing practice precisely through this mechanism [30]. This finding marks the first time that change fatigue has been extended from the concept of “employee psychological experience” in organizational behavior to the category of “upstream risk factors” in the field of patient safety. In other words, change fatigue is not only a professional health issue for individual nurses but also a “hidden eroder” that can indirectly lead to preventable injuries—such as stress‐related injuries—by compromising the quality of preventive behavior implementation. From the perspective of patient safety science, this suggests that hospital quality management systems should incorporate nurses’ psychological resource depletion into routine monitoring and early warning indicators, rather than focusing solely on post hoc statistics of adverse outcomes. A systematic review by Beaulieu et al. [14]on change fatigue in the nursing field noted that change fatigue leads to emotional detachment, decreased professional vitality, and passive adaptation to change among nurses—all of which may undermine the quality of nursing practice. A qualitative study by Duan et al. [31]on Canadian nurses found that change fatigue is a core factor leading to decreased work engagement among nurses, and that nurses explicitly associate change fatigue with patient safety risks.

The detrimental effect of change fatigue on preventive behaviors also draws attention to a neglected public health issue: the “health cost” of organizational change. China’s healthcare system is currently undergoing rapid transformation—DRG/DIP payment reforms, nursing performance system overhauls, and smart hospital construction are all advancing. While these changes improve systemic efficiency, they simultaneously impose sustained psychological demands on frontline nurses [29]. During periods of rapid and continuous change, change fatigue emerges as a critical factor determining the success of organizational transformation. Our findings suggest that change fatigue is not merely an individual psychological phenomenon but also a potential public health risk—by eroding the quality of nurses’ preventive care, it increases the likelihood of preventable conditions such as PIs, thereby prolonging hospital stays and consuming additional resources, thus perpetuating a negative cycle of “organizational change ⟶ individual depletion ⟶ prevention failure ⟶ adverse outcomes ⟶ resource waste.”

### 4.5. Nursing Management Implications Based on the “Resource Conversion–Depletion Buffering” Chain

This study offers several practical implications for nursing management and hospital administration.

At the organizational level, our findings suggest that ensuring adequate staffing, equipment availability, and PI‐related training opportunities may support nurses’ preventive behaviors. Because POS was directly associated with PI care behaviors (*β* = 0.387), nursing managers should pay attention to the tangible resources and emotional support they provide to frontline nurses. Specifically, hospitals could conduct routine assessments of nurses’ POS to identify units or departments where support is inadequate, and target resource allocation accordingly.

At the individual level, strengthening nurses’ self‐efficacy in PI management may be a promising strategy. Given the strong association between self‐efficacy and PI care behaviors (*β* = 0.462), nursing managers could implement progressive goal‐setting, mastery experiences through simulation training, and peer support programs to enhance nurses’ confidence in performing PI prevention. These strategies are particularly relevant for newly recruited nurses or those with limited PI care experience.

At the occupational health level, routine monitoring and tiered support for change fatigue during organizational change may be considered. Because change fatigue was negatively associated with PI care behaviors (*β* = −0.187), hospitals implementing major reforms (e.g., DRG/DIP payment reform, nursing performance system overhauls, smart hospital construction) could concurrently monitor nurses’ change fatigue and provide transparent communication, reasonable transition periods, and psychological support resources. Tiered interventions could be offered based on the severity of fatigue detected.

At the policy level, whether organizational health indicators—such as nurses’ POS and change fatigue—could inform future quality and safety assessment deserves prospective evaluation. However, the present findings provide preliminary rather than definitive support for this direction. While the present findings provide preliminary rather than definitive support for this direction, these findings raise the possibility that quality and safety management might, in future, complement outcome‐oriented approaches (e.g., counting PI incidence) with prospective monitoring of organizational processes and nurses’ psychological resources. However, this possibility requires further prospective evaluation before any policy recommendation can be made. This aligns with international calls for systematic organizational investment in PI prevention.

## 5. Conclusion

Drawing on the JD‐R model and the COR theory, this study examined the relationship between POS and nurses’ PI care behaviors, along with the serial mediating roles of self‐efficacy and change fatigue. The results indicate that POS was directly associated with PI care behaviors and indirectly associated with them through the sequential pathway of “self‐efficacy ⟶ change fatigue.” These associations are consistent with, but do not establish, the hypothesized causal ordering.

Theoretically, this study extends the integrated application of the JD‐R and COR frameworks in nursing behavior research by showing how external organizational resources may be converted into internal psychological capital, thereby buffering occupational depletion and promoting clinical behaviors. Practically, nursing managers should strengthen organizational support through optimized staffing, training opportunities, and emotional care, and should monitor change fatigue as an occupational health risk during organizational transitions.

This study has several limitations. The cross‐sectional design precludes causal inference; future studies could adopt longitudinal or experience sampling designs, as exemplified by Shahzad et al. [32], who used two‐wave data with a three‐month lag to examine a sequential mediation pathway. All variables were self‐reported; although Harman’s single‐factor test and a single‐factor CFA indicated that common‐method variance was not a major concern, future studies should incorporate objective PI‐care indicators and multisource assessments. The sample was drawn exclusively from three tertiary‐level A hospitals in Xinjiang, limiting generalizability. Although skewness and kurtosis were acceptable, the Mardia test indicated multivariate non‐normality; future studies may consider robust or Bayesian estimators when distributions are more markedly non‐normal. Because the hypothesized model was saturated, it had no degrees of freedom for global fit testing. Finally, although adjustment for years of service and PI‐care experience yielded essentially unchanged estimates, hospital site was significantly associated with self‐efficacy, change fatigue, and PI care behaviors, and the focal path coefficients were attenuated after adjustment; because only three hospitals were included, multilevel modeling was not feasible, and future multicenter studies with more sites should use multilevel modeling to partition between‐ and within‐hospital variance.

## Funding

This research received no specific grant from any funding agency in the public, commercial, or not‐for‐profit sectors.

## Disclosure

All authors approved the final version to be published.

## Conflicts of Interest

The authors declare no conflicts of interest.

## Supporting Information

Additional supporting information can be found online in the Supporting Information section.

## Supporting information


**Supporting Information** STROBE_checklist_cross‐sectional.

## Data Availability

The data that support the findings of this study are available from the corresponding author upon reasonable request.
